# Determinants of research use in clinical decision making among physical therapists providing services post-stroke: a cross-sectional study

**DOI:** 10.1186/1748-5908-5-77

**Published:** 2010-10-14

**Authors:** Nancy M Salbach, Sara JT Guilcher, Susan B Jaglal, David A Davis

**Affiliations:** 1 Department of Physical Therapy, Faculty of Medicine, University of Toronto, 160-500 University Avenue, Toronto, Ontario, M5G 1V7 Canada; 2 Department of Health Policy, Management and Evaluation, Faculty of Medicine, University of Toronto, Health Sciences Building, 155 College Street, Suite 425, Toronto, Ontario, M5T 3M6 Canada; 3 Association of American Medical Colleges, 2450 N Street, N.W., Washington, D.C., 20037-1127 USA

## Abstract

**Background:**

Despite evidence of the benefits of research use in post-acute stroke rehabilitation where compliance with clinical practice guidelines has been associated with functional recovery and patient satisfaction, the rate of reliance on the research literature in clinical decision making among physical therapists is low. More research examining factors that motivate physical therapists to consider research findings in neurological practice is needed to inform efforts to intervene. The objective of this study was to identify practitioner, organizational, and research characteristics associated with research use among physical therapists providing services post-stroke.

**Methods:**

A cross-sectional mail survey of physical therapists providing services to people with stroke in Ontario, Canada was conducted. The survey questionnaire contained items to evaluate practitioner and organizational characteristics and perceptions of research considered to influence evidence-based practice (EBP), as well as the frequency of using research evidence in clinical decision making in a typical month. Ordinal regression was used to identify factors associated with research use.

**Results:**

The percentage of respondents reporting research use in clinical decision making 0 to 1, 2 to 5, or 6+ times in a typical month was 33.8%, 52.9%, and 13.3%, respectively (n = 263). Academic preparation in the principles of EBP, research participation, service as a clinical instructor, self-efficacy to implement EBP, a positive attitude towards research, perceived organizational support of research use, and Internet access to bibliographic databases at work were each associated with research use and placed in the final regression model. In the final model (n = 244), academic preparation in EBP, EBP self-efficacy, agreement that research findings are useful, and research participation each remained significantly associated with research use after adjusting for the effects of the other variables in the model.

**Conclusions:**

A third of therapists rarely use research evidence in clinical decision making. Education in the principles of EBP, EBP self-efficacy, a positive attitude towards research, and involvement in research at work may promote research use in neurological physical therapy practice. Future research is needed to confirm these findings and to determine the type of research participation that may promote research use.

## Background

Evidence-based medicine has been described as 'the conscientious, explicit, and judicious use of current best evidence in making decisions about the care of individual patients' [1]. Numerous perceived benefits of evidence-based practice (EBP), including improvement to the work environment, increased professional accountability, ensuring the future of the profession, improved efficiency of service delivery, and compliance with regulatory agencies or quality assurance initiatives in the workplace, may lead healthcare professionals to incorporate research evidence into clinical practice [2]. A patient-centered motivation for appropriately applying findings from rigorously conducted research in clinical decision making is to improve the quality of healthcare services and patient outcomes. There is empirical evidence to support these latter benefits in post-acute stroke rehabilitation, wherein the degree of compliance with a clinical practice guideline has been associated with not only physical recovery [3] but also patient satisfaction [4].

Despite the expected benefits of implementing EBP, a repeated finding of qualitative and survey research is that physical therapists do not readily consult the research literature to inform clinical decision making [5-9]. Few studies, however, have quantified the extent of the problem. A survey [7] of 488 American physical therapists found that a quarter of respondents use research findings in clinical decision making rarely, that is, 0 or 1 time in a typical month, compared to approximately 49% that reported use 2 to 5 times in a typical month. In a survey of 124 Australian physical therapists, 43.9% indicated they either 'never' or 'less often than monthly' integrated research evidence with their expertise [10]. A Canada-wide study of 1,800 rehabilitation clinicians provided convincing evidence that rehabilitation therapists do not routinely apply best practices in the management of urinary incontinence [11], family-centered care [12], and client participation post-stroke [13].

To narrow the knowledge to practice gap, we need to identify factors that exert the greatest influence on research use. The limited research that has been conducted to date has shown that although a high level of training is associated with research use among physical therapists [10], the number of patients seen and hours worked per day, time since graduation, number of physical therapists in the practice setting, access to sources of evidence, or work setting are not [7,9,10]. According to knowledge translation experts, a more comprehensive evaluation of factors should include characteristics of the adopter and the organization, perceptions of the innovation, and readiness to change behavior to better understand the mechanisms driving research use in physical therapy practice [14,15].

The existing literature highlights specific practitioner characteristics -- such as insufficient education in the principles of EBP, and skill and self-efficacy to search, appraise, and apply findings from the research literature in clinical practice -- as substantial barriers to EBP [7,16-18], particularly among older individuals [18,19]. Self-efficacy beliefs, defined as judgments of ability to organize and execute given types of performances [20], are considered a primary influence on decisions to engage in or avoid particular activities or settings. Self-efficacy to implement EBP has been observed to relate to engagement in online searching and reading of the research literature among physical therapists [21], suggesting that it may also be a determinant of research use.

Factors related to the healthcare organization that may restrict physical therapists' engagement in EBP include lack of a mandate (*i.e.*, a written requirement) supporting EBP [18,22] and failure to provide protected time to pursue EBP activities [7,16,18]. Physical therapists in neurological practice report that the supervision of physical therapy students can provide an opportunity to learn about the latest research on a given topic [23]. Limited acceptance of new practices by peers and isolation from peers may represent important setting-specific barriers to implementing new knowledge [24], whereas computer resources and financial support of professional development opportunities are seen as facilitators [25].

Studies highlight perceptions of the available research that may prevent the implementation of EBP. For example, in large surveys, approximately one-third of physical therapists identify the lack of generalizability of research findings to their patient population as a barrier to EBP [7,18]. In qualitative research, some physical therapists have described a lack of trust in research that may limit research use in clinical practice [6].

In summary, research to date investigating barriers to research use has been largely descriptive and has lacked a conceptual or theoretical approach to variable selection. Statistical modeling has been used to evaluate the influence of a small set of factors that has excluded perceptions of the research literature and psychological constructs, such as self-efficacy beliefs, derived from theories of behavior change. As a result, determinants of physical therapists' use of research findings in clinical decision making are not well understood despite the importance of this behavior to ensure the quality of healthcare services delivered in the context of stroke management. Clinical practice guidelines identify the physical therapist as an essential interdisciplinary team member to optimize recovery post-stroke supporting the importance of examining their research use [26,27].

We surveyed 270 Canadian physical therapists providing healthcare services to people with stroke [18]. We have previously reported on factors associated with the frequency at which physical therapists report searching the literature using online bibliographic databases and reading research literature relevant to their clinical practice [21]. These are precursor steps to the use of research evidence in clinical decision making, which is an important and desirable practice. The current paper aims to identify determinants of research use in clinical decision making among physical therapists providing healthcare services to people with stroke. Combined with the results of our previous research, findings of the current study will enhance understanding of what motivates physical therapists to undertake EBP activities and help to guide the development of knowledge translation strategies to increase research-informed healthcare for people with stroke.

## Methods

### Study design

A cross-sectional mail survey was carried out to investigate factors influencing the implementation of EBP among physical therapists providing services to people with stroke [18]. Survey methodology incorporated a modified Dillman [28], three-step approach including a baseline mailing, a postcard thank you/reminder card, and a second mailing of the survey questionnaire to non-responders.

### Participants and sampling

We invited physical therapists in neurological practice treating adults with stroke to participate. Our sampling frame was a mailing list of physical therapists registered with the provincial physical therapy regulatory body in Ontario who indicated they worked in adult neurological practice. We mailed a questionnaire to all individuals on the mailing list. The first item of the questionnaire asked participants whether they provided services to people with stroke. Those who responded 'no' were considered ineligible and were instructed to return the questionnaire with the remaining questions unanswered in a pre-stamped envelope provided. Similarly, people who were eligible but unwilling to participate were asked to return the questionnaire with the remaining items left blank as an indication of their refusal to participate.

### The questionnaire

The survey questionnaire was modeled primarily after the work of Jette *et al. *[7] to evaluate practitioner and organizational characteristics, perceptions of research, and performance of EBP activities. As previously described [18,21], subgroups of items were used to determine education about EBP (three items), attitudes towards and beliefs in EBP (seven items), interest (two items) and perceived role (three items) to engage in EBP, socio-demographic characteristics (age, gender, highest degree obtained and number of years in clinical practice), and professional activities (four items). Physical therapists' self-efficacy to perform the steps of EBP [1,29-31] was measured using a new 12-item scale that yields a total score ranging from 0% to 100% [18]. Higher scores reflect a greater degree of confidence in ability to perform the steps of EBP. We also evaluated organizational characteristics, such as perceived organizational and peer support for EBP (two items), organizational resources and support to promote EBP (six items), and practice and setting characteristics (six items). Four items were used to evaluate perceptions of the relevance and clarity of existing research literature in guiding the treatment of walking limitation, a common result of stroke [32]. Most items were statements with which respondents rated their level of agreement on a five-point Likert scale with response options 'strongly disagree,' 'disagree,' 'neutral,' 'agree,' and 'strongly agree.' The availability of organizational resources was rated as 'yes,' 'no,' or 'do not know.'

We evaluated research use by asking participants to rate how often in a typical month they used the research/professional literature in clinical decision making based on the following response options: 0 to 1, 2 to 5, 6 to 10, 11 to 15, or 16+ times [7].

### Statistical methods

Ordinal logistic regression [33] was used to examine relationships between practitioner, organizational, and research characteristics (*i.e.*, independent variables) and use of the research literature in clinical decision making (*i.e.*, dependent variable). Ordinal regression is used when the number of response categories for the dependent variable exceeds two [34].

Independent variables were re-categorized to form binary variables in the following manner before performing ordinal regression. For items with positively worded statements, we pooled the 'strongly agree' and the 'agree' categories to form an 'agree' category and collapsed the 'neutral,' 'disagree' and 'strongly disagree' categories to form a 'disagree' category. For items with negatively worded statements, we combined the 'strongly disagree' and the 'disagree' categories to form a 'disagree' category and pooled the 'neutral,' 'agree' and 'strongly agree' categories to form an 'agree' category. For items rated as 'yes,' 'no,' or 'do not know,' we combined the 'no' and 'do not know' categories assuming that not knowing about the presence of a resource, for example, would be similar to not having the resource [7].

To ensure reliable estimates of association, we pooled categories of demographic variables with low cell counts [35]. Although participants had provided the percentage of work time spent participating in research, responses were clustered at lower percentages. Thus, we dichotomized the percentage scale to create a yes/no scale wherein 0% = no, 1 to 100% = yes). Finally, the high frequency response categories for research use were pooled due to low frequency of endorsement yielding a three-category dependent variable of research use 0 to 1, 2 to 5, and 6+ times in a typical month.

We conducted ordinal instead of logistic regression to minimize information lost in using a two-category outcome [36]. Ordinal regression is based on a proportional odds assumption; that is, the odds of a unit increase in the dependent variable is considered to be the same across categories [33]. This means that the odds of using research 2 to 5 or 6+ times compared to 0 to 1 time in a typical month is assumed to be the same as the odds of research use 6+ times compared to 0 to 1 time or 2 to 5 times. A non-significant score test result verifies the proportional odds assumption [34].

In ordinal regression, the c-statistic is used as a measure of the discriminative power of the model [35]. A value for the c-statistic of between 0.5 and 1.0 is desired, as higher values reflect a better ability of the model to discriminate individuals based on their level of research use in clinical decision making [35]. Finally, the assumed linear relationship between EBP self-efficacy and the logit of the dependent variable was confirmed and multicollinearity ruled out through an examination of variance inflation factor values [35].

The approach to analysis was exploratory given the paucity of research in this area. We took a blocked modeling approach and first entered subgroups of independent variables into separate models with research use as the dependent variable [35]. For example, variables for the three items used to evaluate education in EBP were created and entered as a block into a model with research use. For each independent variable in the ordinal regression model, we examined the odds ratio (OR) for every pair of categories of research use (*i.e.*, 2 to 5 times versus 0 to 1 time, 6+ times versus 0 to 1 time and 6+ times versus 2 to 5 times). If a variable was significantly associated with research use within each block (*i.e.*, 95% confidence interval (CI) excluded 1) for at least one of the three comparisons, we included it in the final multivariable model and reported ORs and associated 95% CIs [35]. We used a t-test to determine whether the average rating of EBP self-efficacy was higher among participants with academic preparation in EBP compared to those without it. Statistical analyses were performed using SAS version 9.1 (SAS Institute, Cary, NC). The Research Ethics Board at the University of Toronto provided approval for this study.

## Results

We mailed the questionnaire to 1,155 physical therapists and, of these mailings, 43 envelopes (3.7%) were returned to sender. Of the remaining 1,112 mailings, 702 individuals (63.1%) returned a questionnaire. Among these responders, 334 (47.6%) were eligible to participate in the study. Of the eligible respondents, 270 (80.8%) returned a completed questionnaire and 64 people (19.2%) refused to participate. We analyzed data from the final sample of 270 physical therapists.

We describe respondents and their practice settings in Table 1. The mean age of participants was 40 years (SD = 10, range 23 to 68 years). The majority of respondents were women (88.8%) and 76.9% had obtained a Bachelors degree as their highest degree. Almost half of participants (45.4%) had more than 15 years of practice experience. Work settings were most commonly teaching hospitals (67.3%) and urban (60.9%).

**Table 1:** Characteristics of participants and practice settings (n = 270)

Characteristic	n	%
Age in years		
20 to 29	40	14.9
30 to 39	93	34.7
40 to 49	75	28.0
50+	60	22.4

Gender		
Male	30	11.2
Female	239	88.8

Highest degree held		
Certificate/Diploma	30	11.4
Bachelors	203	76.9
Masters	31	11.7

Years in clinical practice		
<5	40	14.9
5 to 10	59	21.9
11 to 15	48	17.8
>15	122	45.4

Participation in research at work		
No	182	67.9
Yes	86	32.1

Hours worked per week		
<20	28	10.4
20 to 30	51	19.0
31 to 40	154	57.5
>40	35	13.1

Type of practice setting		
Acute care hospital	106	39.6
Rehabilitation hospital	43	16.0
Long-term care facility	13	4.9
Complex continuing care	10	3.7
Community health centre	3	1.1
Community care access centre	14	5.2
Home visiting agency	17	6.3
Private practice/clinic	28	10.5
University/Educational institution	1	0.4
Other	33	12.3

The percentage of respondents who reported using the research literature in clinical decision making at different frequencies in a typical month (n = 263) was: 0 to 1 time (33.8%), 2 to 5 times (52.9%), 6 to 10 times (6.8%), 11 to 15 times (2.7%), 16+ times (3.8%).

### Blocked modeling

Factors that were associated with research use in each block of independent variables included academic preparation in EBP, EBP self-efficacy, disagreement that there is a divide between research and clinical practice, agreement that literature and research findings are useful in daily practice, serving as a clinical instructor, research participation, perceived facility support of research use, and Internet access to bibliographic databases at work. Table 2 presents the descriptive cross-tabulation of these factors with the three levels of research use. Table 3 provides the ORs and 95% CIs for associations observed at the blocked modeling stage. The two factors most strongly associated with research use based on the magnitude of the lower limit of the 95% CI were research participation and EBP self-efficacy. Physical therapists who reported involvement in research activities at work were 5.3 times more likely than therapists who did not participate in research activities to use research literature 6+ times compared to 0 to 1 time in a typical month (OR = 5.3, 95% CI 2.2 to 13.9). Physical therapists with high ratings of EBP self-efficacy were 3.9 times more likely than therapists who rated their self-efficacy 20% lower to use research literature in clinical decision making 6+ times compared to 0 to 1 time a month (OR = 3.9, 95% CI 2.1 to 7.4). The average rating of EBP self-efficacy was 70.3% among respondents with academic preparation in the foundations of EBP compared to 57.7% among therapists without this preparation. The mean difference of 12.6% was statistically significant (95% CI 9.4 to 15.8%, p < 0.0001).

**Table 2:** Cross-tabulation of determinants with research use

Factor	n	Level	Frequency of research use in a typical month		
			
			0 to 1 time	2 to 5 times	6+ times
Academic preparation in EBP, No. (%)	259	No	55 (21.2)	77 (29.7)	10 (3.9)
		Yes	32 (12.4)	60 (23.2)	25 (9.6)

EBP self-efficacy, Mean (SD)	264		57.3 (14.5)	65.5 (13.0)	70.1 (14.4)

Attitude: there is a divide between research and practice, No. (%)	258	No	9 (3.5)	31 (12.0)	12 (4.6)
		Yes	78 (30.2)	105 (40.7)	23 (8.9)

Attitude: literature and research findings are useful in daily practice, No. (%)	257	No	33 (12.8)	24 (9.3)	1 (0.4)
		Yes	52 (20.2)	113 (44.0)	34 (13.2)

Research participation, No. (%)	261	No	70 (26.8)	91 (34.9)	14 (5.4)
		Yes	18 (6.9)	48 (18.4)	20 (7.7)

Clinical instructor, No. (%)	262	No	34 (13.0)	31 (11.8)	11 (4.2)
		Yes	54 (20.6)	108 (41.2)	24 (9.2)

Perceived facility support of research use in practice, No. (%)	260	No	33 (12.7)	28 (10.8)	4 (1.5)
		Yes	53 (20.4)	111 (42.7)	31 (11.9)

Internet access to bibliographic databases at work, No. (%)	260	No	29 (11.2)	20 (7.7)	2 (0.8)
		Yes	58 (22.3)	118 (45.4)	33 (12.7)

Abbreviations: EBP, evidence-based practice.

**Table 3:** Factors associated with research use after blocked modeling

Factor	Level	Frequency of research use in a typical month Block odds ratio (95% CI)		
		
		2 to 5 versus 0 to 1 times	6+ versus 0 to 1 times	6+ versus 2 to 5 times
Academic preparation in EBP	No	Reference		
	Yes	1.2 (0.6 to 2.6)	**4.0 (1.3 to 12.7)**	**3.2 (1.1 to 9.7)**

EBP self-efficacy	20% difference	**2.4 (1.6 to 3.6)**	**3.9 (2.1 to 7.4)**	1.7 (0.9 to 3.0)

Attitude: there is a divide between research and practice	No	Reference		
	Yes	0.6 (0.2 to 1.3)	**0.3 (0.1 to 0.9)**	0.5 (0.2 to 1.3)

Attitude: literature and research findings are useful in daily practice	No	Reference		
	Yes	**2.2 (1.1 to 4.6)**	**12.6 (1.5 to 103.2)**	5.6 (0.7 to 46.1)

Research participation	No	Reference		
	Yes	**2.1 (1.1 to 4.0)**	**5.3 (2.2 to 13.9)**	**2.5 (1.1 to 5.6)**

Clinical instructor	No	Reference		
	Yes	**2.1 (1.2 to 3.9)**	1.4 (0.6 to 3.5)	0.7 (0.3 to 1.6)

Perceived facility support of research use in practice	No	Reference		
	Yes	**2.3 (1.3 to 4.2)**	**4.6 (1.5 to 14.1)**	2.0 (0.6 to 6.1)

Internet access to bibliographic databases at work	No	Reference		
	Yes	**3.5 (1.6 to 7.9)**	**9.5 (1.8 to 48.8)**	2.7 (0.5 to 13.6)

Abbreviations: EBP, evidence-based practice.

### Final model

The final multivariable model was based on a complete dataset from 244 participants due to missing data on select questionnaire items. Academic preparation in EBP, EBP self-efficacy, agreement that research findings are useful and research participation each remained significantly associated with research use in clinical decision making after adjusting for the effects of the other variables in the model (Table 4).

**Table 4:** Final ordinal regression model* (n = 244)

Factor	Level	Frequency of research use in a typical month Odds ratio (95% CI)		
		
		2 to 5 versus 0 to 1 times	6+ versus 0 to 1 times	6+ versus 2 to 5 times
Academic preparation in EBP	No	Reference		
	Yes	0.7 (0.3 to 1.4)	2.9 (0.9 to 8.9)	**4.1 (1.5 to 11.1)**

EBP self-efficacy	20% difference	**2.2 (1.3 to 3.6)**	2.0 (0.9 to 4.6)	0.9 (0.5 to 1.9)

Attitude: there is a divide between research and practice	No	Reference		
	Yes	0.6 (0.3 to 1.5)	0.4 (0.1 to 1.3)	0.7 (0.3 to 1.7)

Attitude: literature and research findings are useful in daily practice	No	Reference		
	Yes	**2.7 (1.3 to 5.5)**	**14.2 (1.7 to 118.5)**	5.2 (0.6 to 42.2)

Research participation	No	Reference		
	Yes	1.5 (0.8 to 3.1)	**4.3 (1.6 to 11.7)**	**2.8 (1.2 to 6.7)**

Clinical instructor	No	Reference		
	Yes	1.6 (0.8 to 3.3)	0.9 (0.3 to 2.7)	0.5 (0.2 to 1.5)

Perceived facility support of research use in practice	No	Reference		
	Yes	1.4 (0.7 to 2.9)	2.4 (0.6 to 10.0)	1.7 (0.4 to 6.7)

Internet access to bibliographic databases at work	No	Reference		
	Yes	1.8 (0.8 to 4.2)	2.9 (0.5 to 16.5)	1.6 (0.3 to 8.6)

Abbreviations: EBP, evidence-based practice.

*Score test (p > 0.05), c-statistic = 0.75.

### Research use 2 to 5 times versus 0 to 1 time in a month

Agreement that research findings are useful (OR = 2.7, 95% CI 1.3 to 5.5) and EBP self-efficacy (OR = 2.2, 95% CI 1.3 to 3.6) were associated with research use 2 to 5 times versus 0 to 1 time a month. Physical therapists who perceived research as useful in daily practice were 2.7 times more likely than those who did not perceive research as useful to use research 2 to 5 times compared to 0 to 1 time in a month after adjusting for the remaining variables in the model. Physical therapists with high ratings of EBP self-efficacy were 2.2 times more likely than peers who rated their self-efficacy 20% lower to use research literature 2 to 5 times in a typical month compared to 0 to 1 time (OR = 2.2, 95% CI 1.3 to 3.6) after adjusting for the remaining variables in the model.

### Research use 6+ times versus 0 to 1 time in a month

Physical therapists who agreed that research findings are useful were 14.2 times more likely than those who did not agree to use research literature 6+ times versus 0 to 1 time a month after adjusting for the remaining variables in the model (OR = 14.2, 95% CI 1.7 to 118.5). Therapists who reported participating in research were 4.3 times more likely than those who did not participate to use research literature 6+ times versus 0 to 1 time a month (OR = 4.3, 95% CI 1.6 to 11.7) after adjusting for the remaining variables in the model.

### Research use 6+ times versus 2 to 5 times in a month

Factors that were associated with research use 6+ compared to 2 to 5 times included academic preparation in EBP (OR = 4.1, 95% CI 1.5 to 11.1) and research participation (OR = 2.8, 95% CI 1.2 to 6.7). Physical therapists with academic preparation in EBP were 4.1 times more likely than those without to use research in clinical decision making 6+ compared to 2 to 5 times a month. Further, therapists who participated in research were 2.8 times more likely compared to those who did not participate to use research 6+ compared to 2 to 5 times a month.

## Discussion

In this exploratory study, we observed a moderate rate of research use in clinical decision making and identified a number of factors associated with research use among Canadian physical therapists providing healthcare services to people with stroke. Results indicate that a third of physical therapists use research findings in clinical decision making rarely or not at all, and only 13.3% of therapists integrate research in clinical decision making 6+ times in a typical month. The actual rate of research use in clinical decision making may be lower than that observed in this study for two reasons: participants may be more engaged in EBP activity than non-responders and some may have wished to provide socially desirable answers.

The frequency of research use that we observed, however, was slightly lower than corresponding rates reported following a survey of American physical therapists where approximately 25% of therapists used research 0 to 1 time and 26% used research 6+ times in a typical month. Sampling physical therapist members of the American Physical Therapy Association, as well as the inclusion of participants with doctoral-level training in the American study, may explain differences between study results, particularly given our finding that education in EBP is associated with self-reported research use.

It is important to understand how the correlates of research use identified in the current study complement our previous analysis of antecedent behaviors such as searching and reading the research literature [21], and investigations of therapists' information sources [23] and perceptions of the research literature [37]. Academic preparation in the principles of EBP appears to distinguish high-level users from moderate-level users of research in clinical decision making. Our research findings in this and in previous studies support relationships between academic preparation in EBP and self-efficacy to implement EBP, and between EBP self-efficacy and performance of three EBP behaviors: online searching and reading of the research literature, and use of research evidence in clinical decision making. In light of the four strategies for increasing self-efficacy [20], and the theoretical [20] and empirically supported role of self-efficacy as a determinant of work-related performance [38], it is likely that increasing self-efficacy is one of the biological mechanisms through which education impacts healthcare professionals' engagement in EBP behaviors. Healthcare professional programs that provide opportunities through class assignments and clinical internships for students to experience success in searching and appraising the research literature and incorporating it in clinical decision making (*i.e.*, mastery experiences) are likely to boost students' self-efficacy and performance of these activities following graduation. Postgraduate education targeting competence in EBP that provide opportunities for applying skills to current patient scenarios in the clinical practice environment may be more likely than interventions carried out external to the practice environment to elicit a change in subsequent clinical behavior [39] due to the importance of contextual factors in either facilitating or impeding work performance [38].

Although we measured self-efficacy of individual practitioners in the current study, related constructs that are particularly relevant to effective functioning of interdisciplinary stroke teams in implementing EBP include collective efficacy [40], the shared belief that the group can achieve a collective outcome, and group-derived efficacy [41], the extent to which individuals see the group as assisting the attainment of their personal goals for change or achievement. Individual and group-related self-efficacy represent important variables to consider in future investigations of EBP in the context of stroke team functioning.

Attitude is a construct closely related to self-efficacy beliefs, and our findings show that attitudes can help to explain the implementation of EBP. Although we modeled therapists' responses to a number of statements evaluating attitudes towards EBP, such as EBP 'is necessary,' 'improves the quality of care,' and 'helps with decision making,' only the perceived usefulness of research findings in daily practice corresponded to therapists' degree of research use in clinical decision making. This particular attitude discriminated both moderate- and high-level users of research from low-level users who reported using research none or one time in a typical month in addition to relating to frequency of reading the research literature [21]. Entry-level professional training programs, healthcare teams, and postgraduate education interventions that effectively convince practitioners of how the new knowledge or innovation will specifically improve their daily practice may promote research use in clinical practice.

Participation in research activities for some percentage of work time seems particularly important as it has been associated with searching, reading [21], and, in the current study, using research in clinical decision making. The relationship with research use has also been observed in the nursing literature [15,42]. Interestingly, 74% of the 86 therapists participating in research did so for only a small percentage (1 to 5%) of their work time [21]. Elucidating the nature of this activity and its influence on research use may reveal a time-efficient strategy for promoting research use in clinical practice. It is reasonable that research participation, positive attitudes, and self-efficacy to implement EBP are inter-related and important factors that facilitate the incorporation of research findings into clinical decision making. That both EBP self-efficacy and research participation remained significant in the final model after adjusting for the effects of all of the other variables underscores their potential importance for engagement in EBP.

Finally, the significant association observed between perceived facility support of research use and therapists' research use in clinical decision making deserves note. Perceived facility support was independently associated with three EBP behaviors: searching, reading [21], and, in this study, using research findings in clinical decision making, but did not remain significant after adjusting for other variables in the final model. In addition to perceived facility support, we adjusted for research participation in the final multivariable model, which may have explained similar variance in research use as perceived facility support. Further research is needed to understand the specific nature of this support and the potential interactions between such organization-level variables as perceived facility support of EBP, provision of EBP resources, and research participation in order to identify optimal organizational strategies that facilitate research use in clinical practice.

Overall, this study revealed significant and modifiable correlates of research use at the level of the practitioner (*i.e.*, academic preparation, attitude towards research, self-efficacy, research participation, being a clinical instructor,) and the organization (*i.e.*, Internet access, facility support of research use), underscoring that efforts to promote evidence-based physical therapy practice cannot target the physical therapist alone.

## Limitations

The cross-sectional study design limits inferences that the associations observed are causal in nature. Findings may be limited to the Canadian context where the highest entry-level professional physical therapy programs are at the Masters level. The strengths of this research are the use of conceptual and behavior change frameworks to identify potentially influential variables related to research use. Participants were sampled from the provincial registry of physical therapists and not from membership lists of professional associations, which supports the generalizability of results to physical therapists in neurological practice. Finally, the use of a three-category dependent variable in statistical modeling and comparison of pairs of dependent variable categories optimized identification of variables most closely linked to specific levels of research use.

## Summary

Although a third of physical therapists rarely use research evidence in clinical decision making, results suggest a number of potential mechanisms through which to improve research use among physical therapists providing services to people with stroke. Future investigations of EBP should focus on the effects of academic training, self-efficacy beliefs, perceptions of the usefulness of research evidence in daily clinical practice, participation in research activities at work, supervision of trainees, organizational support of research use, and provision of Internet access to bibliographic databases at work.

## Competing interests

The authors declare that they have no competing interests.

## Authors' contributions

NMS conceived of, designed, and carried out the study, planned the statistical analysis, and drafted the manuscript. SJTG helped plan the analysis, performed the statistical analysis, and assisted in drafting the manuscript. SBJ and DAD contributed to conceiving the study. All authors read, provided critical input on and approved the final manuscript.
